# Diabetic Emotional Burden and Post Covid-19 Health Care Services for Diabetic Patient: A New Dimension of Access to Healthcare

**DOI:** 10.1900/RDS.2022.18.187

**Published:** 2022-12-31

**Authors:** Suresh Chandra Akula, Pritpal Singh, Waseem Ul Hameed, Rupjyoti Talukdar, Shalini Patlolla, Muhammad Murad

**Affiliations:** 1 Mittal school of business, Lovely Professional University, G.T. Road, Phagwara, Punjab144411, India,; 2 Centre of Excellence for Islamic Finance and Social Equity (CEIFSE), Department of Islamic and Conventional Banking (DICB), Institute of Business Management and Administrative Sciences (IBMAS), The Islamia University of Bahawalpur (IUB), Pakistan,; 3 Asian Institute of Gastroenterology (AIG), Hyderabad, India,; 4 Othman Yeop Abdullah Graduate School of Business, Universiti Utara Malaysia (UUM), Malaysia.

**Keywords:** diabetic emotional burden, health care access ·healthcare system, doctor and nurse distress, interpersonal distress

## Abstract

In the era of post-covid-19, new trends have emerged in healthcare services and healthcare access. Diabetic patients are more concerned about their health care services as social awareness is increased. This study aims to investigate the role of diabetic emotional burden and healthcare services as a moderator in the relationship between interpersonal distress, physician & nurse distress, and access to healthcare. The population of this study was the patients with diabetes in different public and private hospitals from Kerala state of India. The study concludes diabetic emotional burden and health care services positively moderate the relationship between interpersonal distress and access to healthcare. This research is a contribution to knowledge as no study earlier was conducted to discuss this gap in the literature. This study has practical and theoretical implications concerned to improve the access to the healthcare system for diabetic patients in Kerala and the rest of the world.

## 1 Introduction

Diabetes patients are suffering from different kinds of problems related to the health care services in India [1]. The problem is related to the emotional and cognitive distress that is a hurdle in the way of treatment of diabetes patients [2]. The number of diabetes patients is increasing, similarly, the public and private hospital sector is increasing to provide better services to diabetes patients [3]. In the era of post-covid-19, new trends are developed for better hospitality to patients with diabetes by the different healthcare systems [1]. It is due to the reason that the patients are now living at distance, and they have required the appropriate health services for their fatal diseases. The special hospital working for diabetes patients is looking forward to adopting new technology and providing better services for the patients with diabetes [4]. In this way, after covid-19 the traditional ways of dealing with patients with diabetes are decreased and eliminated, but new and alternative ways are required to be developed for the better health condition of the patients [5].

A better healthcare system is important for the improvement in the life of a diabetes patient [6]. When the state government is providing a better healthcare system with the help of public and private sector hospitals, there is a need to improve this system for the better health of the patients [7]. The patients have different kind of emotional distress and emotional burden that is not right for patients with diabetes [8]. Similarly, the distance between physicians and a nurse is critical for patients with diabetes because they want systematic and friendly behavior from these authorities when in hospitals [9]. The patient that is emotionally fine and they are developing their ability for improved understanding with the doctors, these patients are getting better services related to their health [10]. In the earlier studies, there was little focus on this area of research to understand the role of emotional distress and health care service in access to the healthcare system by patients of diabetes [1,3,6]. In this way, emotional distress and the distress of physicians and nurse is critical to understanding access to the health care system for the patients [11].

The theoretical framework of the study is developed on the guidelines of earlier studies that are not conducted on the significant area of research discussed in this study. This study aims to understand the role of emotional distress and health care service as moderators of the relationship between interpersonal distress, physician and nurse distress, and access to healthcare. This is the contribution of this study to the knowledge because no earlier study was conducted to discuss this area of research effectively and contribute to the knowledge. This objective of the study makes it novel because a new dimension of research is conducted to contribute to the literature for the improvement of the health condition of diabetes patients.

**Figure 1.**
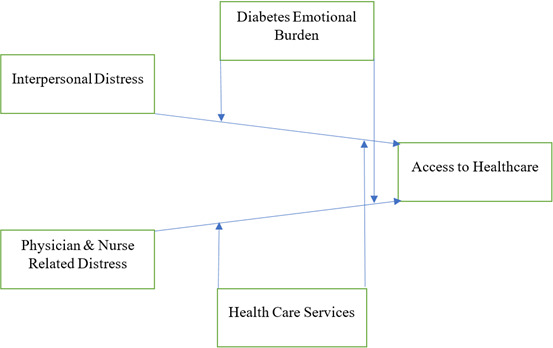
Study model

This study is significant because it is designed to provide theoretical as well as practical implications critically considered for the improvement of the health condition of diabetes in patients. In this way, it must be understood that diabetes patients are provided with an effective healthcare system to improve their health by eliminating the doctor and related distress [12,13]. Similarly, this study points out there is the critical role of emotional burden distress in the improvement of the life of diabetes patients. Meanwhile, this study also provides future direction for the comic studies to improve the open access to the health care system by diabetes patients in the era of post-pandemic. This novelty of the study makes it effective and worthy for the critical condition and improvement in the life of diabetes patients all over the world. [Figure 1]

## 2 Literature review

### 2.1 Role of interpersonal distress and physician & nurse related distress in access to healthcare

In modern times, the access of patients to the healthcare system and services is critical to understand because the changing dynamics and the different healthcare systems in the public and private sectors are providing different opportunities for the people [14]. People with diabetes are more concerned about their health service system because they believe hand without a proper health service system [15], it would be difficult for them to get survival the critical disease.

Similarly, the individual that is in working in different public and private healthcare systems is responsible to manage the activities and appointments of diabetes patients [5,15]. Indeed, every patient wants stability because he is facing a different kind of psychological problems along with the disease. It must be understood that diabetes patients must be provided with an effective health care system and they should get better services when they were the different public or private sector hospitals [5]. It is the core requirement of the healthcare system to provide appropriate facilities on time to get the patients out of different kinds of distress that are critical and problematic for these patients. Similarly, in America, public and private sector hospitals are designed to provide better health services to diabetes patients because they are suffering from psychological problems [2,5]. The patients who believe that doctor is not fair to them and the attitude of the nurse is not supportive, these patients are left wrong in from one hospital to another to get better services [1,4]. It is also noted that in different hospitals the government services are safe but the appropriate and specific services are required by the patient because they are different from the diseases and they have a different set of information. In India, a large number of diabetes patients are not treated because they are not provided with related information about the healthcare management system and information about their diseases [9]. Significantly, the patients that are not supported and treated when by the nurse and doctors, these patients avoid visiting the hospital because they believe that the hospital is not a place of better services but it is a place of humiliation [12,13]. In this way, the focus should be on the improvement of the relationship between the patients and the doctors to provide better health services to the patients in the public and private sector hospitals. The appropriate health services would lead the patients to visit again and again and get better facilities from the hospitals with the help of the friendly behavior of the doctors and nurses [4,11].

*H1. There is a relationship between interpersonal distress and access to health care*.

*H2. There is a relationship between physician and nurse-related distress and access to healthcare*.

### 2.2 Moderating relationship of diabetes emotional burden

Emotions are human tools that cannot be diverse from the human personality [16,17]. It is critical to understand that not only the patients with diseases emotional but they are facing different kinds of problems due to these emotions [18]. It must be understood that with the emotional attachment and cognitive problems, different patients with diabetes are suffering from the major problem of not visiting the clinic and hospital for better health services related to their diseases [12,19]. It must be understood that the emotional distress of the patients leads them to not visit the hospital and as a result of it they suffer from different critical problems and death [20]. The majority of the patients with diabetes are suffering from emotional distress because they are not properly motivated and informed about their activities and visit the hospital for health services [5]. Patients with diabetes have a natural stigma and mental fatigue to not with the hospitals if they are not appropriately treated by the doctors and the nurse [21,22]. They must understand that patients who are suffering from different problems related to diabetes must be treated in a way that they should develop an effective cognitive relationship with the institution and hospital [23]. In India, the majority of the patients are treated well and they are emotionally attached to the doctor and nurse, these patients are visiting, again and again, hospitals for better treatment and mental health services [24]. In America and Norway, the core responsibility of the doctor and nurse is to be emotionally attached to the patients and provide them are fans of relaxation and friendship during the treatment [12]. When the patients are emotionally not in any distress, and they are actively involved to get better facilities and services, they go for the word-of-mouth marketing of the doctors and hospitals as well [6,14]. The policy development should be in a way to improve the experience of the patients and the emotional attachment must be done for the proper elimination of emotions and emotional distress from the patients [4,5]. It is also the responsibility of the patients to get things in an attractive way when they are at hospitals, and it must be well attached to their behavior and proportion for visiting the hospital again and again to get better services and facilities [4,20]. The relationship between the doctor and the patients must be friendly and they should develop emotional thoughts to improve the critical situation of the patients [25].

*H3. There is a moderating role of diabetes emotional burden in the relationship between interpersonal distress and access to healthcare*.

*H4. There is a moderating role of diabetes emotional burden in the relationship between physician and nurse-related distress and access to healthcare*.

### 2.3 Moderating relationship of health care services

In modern times, health services are improved with the advancement of technology in the health sector that is provided the opportunity to people for improving their mental and physical health conditions [26,27]. It is critical to understand that the patients that are treated well and provided better health services, get better access to health services in society [28,29]. The American government is working to improve the health sector by providing better facility is related to the health of patients with diabetes, because with the help of better health services they would develop a cognitive affection and a relationship for better treatment from the public or private sector hospitals [5,30-32]. The policy development for better health services should be in a way to attract the patients with diabetes and provide them better services to reduce their problematic infection. Indeed, in India, the development of the hospital sector in improving better health service facilities for patients with diabetes as different special hospitals are working to treat the patients [1,33]. However, the responsibility of the health ministry is not over, but it is to develop the policies and implement them with the help of management to provide better health services to the patients [1-3]. It is because the patients that are suffering from different kinds of health-related distress, when they would be provided with the appropriate opportunities to improve their health conditions, it would be more effective to develop their thought and proper healthcare services from the private and public sector hospitals [7,14]. The patients are required to analyze the health services critically and choose the hospital that is providing better health services for advanced working and treatment [4,34]. The cooperation between the doctor and the patients would be improved with the advanced and better-related facilities would be provided to the patients with diabetes [12]. In the state of Kerala, the government health ministry is working to provide better health-related policies for the health sector improvement and better health condition of diabetes patients [12,35]. It must be understood that the health services are not imported alone, but the services must be done in an effective way to improve the emotional attachment with the patients and better services should be provided for the development of diabetes patients [11]. The patients who believe that better health services are offered by different public and private sector hospitals, these patients are vo concert to get treatment for their effective development [7].

*H5. There is a moderating role of the healthcare system in the relationship between interpersonal distress and access to healthcare*.

*H6. There is a moderating role of the healthcare system in the relationship between physician and nurse related to distress and access to healthcare*.

## 3 Methodology

### 3.1 Questionnaire

This research is based on the quantitative data collected from the target population living in the Indian state of Kerala. The majority of earlier studies on diabetes were conducted on secondary data taken from health journals and reports. However, a few studies were also based on interviews and quantitative data collected on the questionnaire, from the target population. In this manner, this study adopted a quantitative method for data collection as it is an appropriate method to collect the data from a large population on a cluster-based sampling method. The questionnaire was prepared by adapting the scale items from the earlier studies. The scale items for physician & nurse-related distress, interpersonal distress, and diabetes emotional burden were adapted from the study of Thanakwang et al. [2014]. Also, the scale items for healthcare services were adapted from Ismail et al. [2020]. On the other hand, the scale items for access to health care were adapted from Mühlbacher & Juhnke [2013]. The questionnaire was reviewed by the experts in diabetic research to confirm the face validity and integration of the constructs before collecting the data from the target respondents.

### 3.2 Data collection process

The data for this study was collected on a five-point Likert scale questionnaire. The cluster-based sampling technique was adopted for this study because the population was large. In this way, 1500 questionnaires were prepared and the target population was divided into two main clusters on a gender basis. Half of the questionnaires were required to be filled by the women patients, and the other half was supposed to be filled by the men patients. Therefore, 1500 copies of questionnaire were distributed among the respondents. Before data collection, a verbal consent was taken from the respondents and questionnaires were distributed among those participants which were agreed to respond. The researcher visited different public and private hospitals in Kerala and got information about the appointments of diabetes patients. The questionnaires were distributed to the target population and divided into clusters, and a brief introduction to the study was provided. Importantly, all the questions of the respondents were addressed during the response to the questionnaire. After the collection of data, the respondents were apricated for their honest response and contribution to the worth of the study. Moreover, out of 1500, only 980 questionnaires were returned with the correct response and considered for this study.

## 4 Findings

### 4.1 Convergent validity

**Figure 2.**
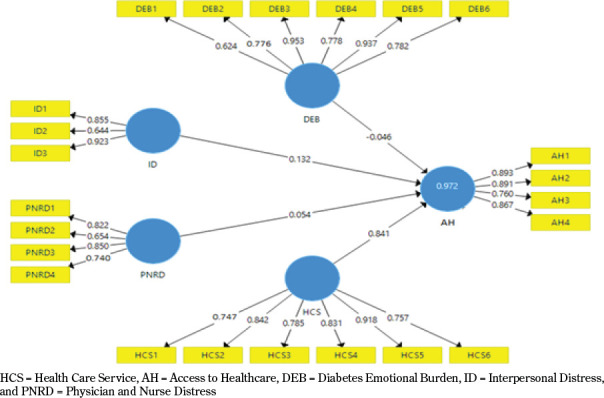
Measurement model

The study’s convergent validity was checked with PLS Software which is useful for the advance and rich in research studies. To begin with, PLS Algorithm calculations were taken to check factor loading, Cronbach’s alpha, compositive reliability, and average variance extracted. The values of factor loadings were not less than 0.60 for each construct in this study. Similarly, the values of composite reliability were not less than 070 as recommended by the study of Henseler & Fassott [2010]. Also, the values of average variance extracted were determined, and the value for each scale item was not less than 0.50 as recommended by Hair Jr et al. [2014]. The results available in Table 1 demonstrate the clear reliability and validity of the scale items [see Figure 2].

**Table 1:** Items, Factor Loadings, Cronbach’s Alpha, Composite Reliability (CR), and Average Variance Extracted (AVE)

Variables		Items	Factor Loadings	Alpha	CR	AVE
Access to Healthcare	The surgery hours are flexible.	AH1	0.893	0.875	0.915	0.730
	The service provider takes a proactive approach with me and agrees check-up appointments or reminds me that an appointment is due.	AH2	0.891			
	My service provider is available around the clock in case of emergencies.	AH3	0.760			
	The health insurer actively supports me.	AH4	0.867			
Diabetes Emotional Burden	Feeling that diabetes controls my life.	DEBI	0.624	0.876	0.905	0.620
	Not feel confident in my day-to-day ability to manage diabetes.	DEB2	0.776			
	Feeling overwhelmed by the demands of living with diabetes.	DEB3	0.953			
	Feeling that I am often failing with my diabetes routine.	DEB4	0.778			
	Feeling that diabetes is taking up too much of my mentol and physical energy every day.	DEB5	0.937			
	Feeling that I am not testing my blood sugars frequently enough and not feeling motivated to keep up my diabetes selfmanagement.	DEB6	0.782			
Health Care Services	The pharmacist is polite and friendly.	HCS1	0.747	0.874	0.906	0.622
	The pharmacist provides medication with a clear drug label and explanation.	HCS2	0.842			
	The pharmacist listens to what I have to say.	HCS3	0.785			
	The pharmacist explains how to take the medications and why it is important to take my medications as directed.	HCS4	0.831			
	The pharmacist is helpful when I have problems with my medications.	HCS5	0.918			
	The operating hours of the pharmacy are satisfactory.	HCS6	0.757			
Interpersonal Distress	Feeling that friends or family don't give me the emotional support that I would like.	ID1	0.855	0.745	0.854	0.666
	Feel that friends or family don't appreciate how difficult living with diabetes can be.	ID2	0.644			
	Feeling that friends or family are not supportive enough of selfcare efforts.	ID3	0.923			
Physician & Nurse Related Distress	Feeling that my doctor or nurse doesn't take my concerns seriously enough.	PNRD1	0.822	0.758	0.814	0.530
	Feeling that my doctor or nurse doesn't give me clear enough directions on how to manage my diabetes.	PNRD2	0.654			
	Feeling that I don't have a doctor or nurse whom I can see regularly enough about my diabetes.	PNRD3	0.850			
	Feeling that my doctor or nurse doesn't know enough about diabetes and diabetes care.	PNRD4	0.740			

HCS = Health Care Service, AH = Access to Healthcare, DEB = Diabetes Emotional Burden, ID = Interpersonal Distress, and PNRD = Physician and Nurse Distress

### 4.2 Discriminant validity

The study utilized Heteritrait-Monotrait [HTMT] method to check the discriminant validity. In this regard, PLS Algorithm calculations were identified. Discriminant validity is to check the distinction between the scale items used for each variable of the study [see Table 2]. According to the values, there is a clear discriminant validity for the scale items because no value of discriminant validity HTMT was greater than 0.90 as recommended by Henseler et al. [2014].

**Table 2.** Discriminant Validity Results – Heteritrait-Monotrait (HTMT)

	AH	DEB	HCS	ID	PNRD
AH					
DEB	0 799				
HCS	0 821	0 752			
ID	0.783	0.738	0.682		
PNRD	0.829	0.832	0.822	0.723	

HCS = Health Care Service, AH = Access to Healthcare, DEB = Diabetes Emotional Burden, ID = Interpersonal Distress, and PNRD = Physician and Nurse Distress

### 4.3 Partial Least Square – Structural Equation Modelling [SME]

**Figure 3.**
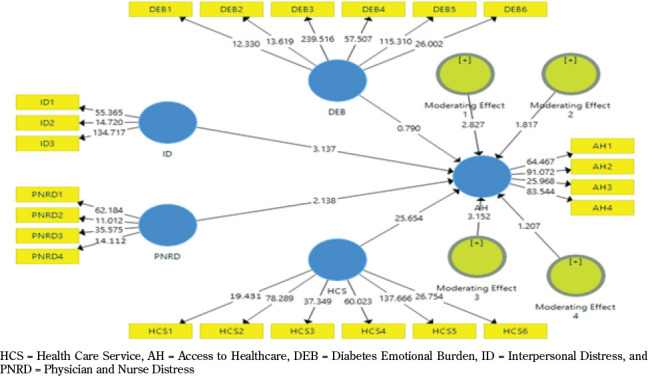
Structural model

In this study, PLS Software was used to determine the relationship between different variables available in the framework of the study [see Figure 3]. Moreover, PLS Bootstrapping calculations were used to test the hypotheses. On the one hand, according to the results of hypothesis 1 [β = 0.132, T= 3.137, and P= 0.002], the relationship between interpersonal distress and access to healthcare is significant and supported. On the other hand, according to the results of hypothesis 2 [β = 0.054, T= 2.138, and P= 0.0033], the relationship between physician and nurse distress and access to healthcare is significant and supported. The results of the path coefficient are available in Table 3.

**Table 3.** Path coefficient

No	Relationship	Beta Value	STDEV	T Statistics	P Values	Remarks
1	ID -> AH	0.132	0.042	3.137	0.002	Supported
2	PNRD -> AH	0.054	0.025	2.138	0.033	Supported

AH = Access to Healthcare, ID = Interpersonal Distress, and PNRD = Physician and Nurse Distress

### 4.4 Moderating effect

**Figure 4.**
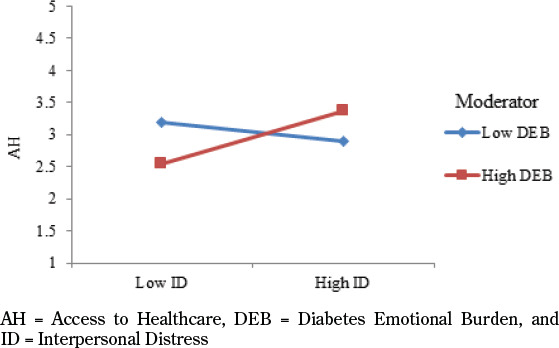
Moderation 1

**Figure 5.**
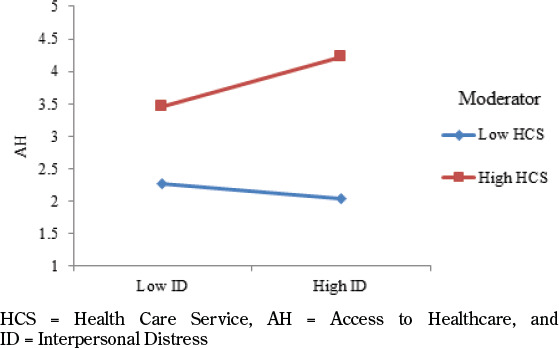
Moderation 3

To check the moderating effects, PLS Software was used to determine the relationship between different variables. Firstly, according to the results of hypothesis 3 [β = 0.274, T= 2.827, and P= 0.005], there is a significant moderating role of diabetes emotional burden in the relationship between interpersonal distress and access to healthcare. Moreover, according to these results, DEB strengthens the relationship between ID and AH [see Figure 4]. Secondly, according to the results of hypothesis 4 [β = 0.200, T= 1.817, and P= 0.070], there is not a significant moderating role of diabetes emotional burden in the relationship between physician and nurse distress and access to healthcare. Thirdly, according to the results of hypothesis 5 [β = 0.242, T= 3.152, and P= 0.002], there is a significant moderating role of health care services in the relationship between interpersonal distress and access to healthcare. Further, according to these results, HCS strengthens the relationship between ID and AH [see Figure 5]. Lastly, according to the results of hypothesis 6 [β = 0.102, T= 1.207, and P= 0.228], there is not a significant moderating role of health care services in the relationship between physician and nurse distress and access to healthcare. The results of moderating effects are available in Table 4.

**Table 4.** Moderation results

No	Relationship	Beta Value	STDEV	T Statistics	P Values	Remarks
1	Moderating Effect 1 -> AH	0.274	0.097	2.827	0.005	Supported
2	Moderating Effect 2 -> AH	0.200	0.110	1.817	0.070	Not Supported
3	Moderating Effect 3 -> AH	0.242	0.077	3.152	0.002	Supported
4	Moderating Effect 4 -> AH	0.102	0.085	1.207	0.228	Not Supported

AH = Access to Healthcare

## 5 Discussion and Conclusions

In this section of the study, the results of the hypotheses’ relationship are discussed. According to the results of hypothesis 1, the relationship between interpersonal distress and access to healthcare is significant and supported. Furthermore, according to the results of hypothesis 2, the relationship between physician and nurse distress and access to healthcare is significant and supported. Indeed, interpersonal distress is leading patients with diabetes to avoid treatment from public or private sector healthcare as discussed by Thanakwang et al. [2014] and Zhang et al. [2008]. It must be understood that with the help of effective policies developed for diabetes patients, the level of stress and interpersonal distress can be decreased for patients with diabetes as demonstrated by Polonsky et al. [2022]. In this way, a sense of positive approach would be developed in the patients and they would be more willing to get the treatment for diabetes in the public and private sector hospitals by getting a sense of friendship from the doctors and nurses as well [36,41]. The doctor that are provided appropriate facilities for diabetes patients, these doctors are developing will a common trust that helps to reduce the infection of diabetes and motivates the patients to get better diabetes treatment [5,12]. The mental problem of any patient including anxiety and psychological barriers in the way of access to the health care system is limiting the patients to reduce their mental problems [20]. Therefore, more focus should be to develop effective policies and implement these policies in the best way for improving the living standard of diabetes patients by providing them appropriate services in the public and private sector health care systems all over the world.

In moderation, according to the results of hypothesis 3, there is a significant moderating role of diabetes emotional burden in the relationship between interpersonal distress and access to healthcare. On the other hand, according to the results of hypothesis 4, there is not a significant moderating role of diabetes emotional burden in the relationship between physician and nurse distress and access to healthcare. At the same time, according to the results of hypothesis 5, there is a significant moderating role of health care services in the relationship between interpersonal distress and access to healthcare. Lastly, according to the results of hypothesis 6, there is not a significant moderating role of health care services in the relationship between physician and nurse distress and access to healthcare. Similarly, diabetes’ emotional burden is not good for the patients because it is creating psychological problems in the world of patients when they are will it to get treatment from the public and private sector hospitals as discussed by Polonsky et al. [2022]. In this regard, the responsibility of the patients is to not get involved in critical psychological problems but to focus on the interpersonal distance to the degree it and get better access to the health care system or services [14,42]. No doubt, better health care services are appropriate for managing the living standard of patients with diabetes when they are not involved in any kind of burden of emotional distress [12]. The responsibility of the management is to work productively and provide better health-related services because when the appropriate related services are provided, the focus of the patients is developed to get these services by reducing the barrier of interpersonal distress [1,2,13,18]. Significantly, in America and Canada, the health service sector is working to improve the level of interpersonal distress and reduce the level of emotional burden distress to provide the best facilities to patients with diabetes as highlighted by M. I. Harris [2000] and Palmer et al. [2022]. In this regard, by providing such kind of facility, the appropriate development is done to improve the lives of diabetes patients.

## 6 Implications

### 6.1 Theoretical implications

This study has significant theoretical applications that are critical to consider for improving the health of diabetic patients. It is critical to understand that for the development of policies there were limited studies earlier to highlight the role of interpersonal distress and emotional distress burden in the development of policies for diabetes patients in the health care system. In this way, this study is a significant contribution to the literature because the gap in the literature was not addressed by any significant study earlier. Importantly, the moderating role of diabetes emotional burden in the relationship between interpersonal distance and access to the health service is a contribution of this study to the literature. Moreover, the moderating role of healthcare service in the relationship between interpersonal distance and access to the health service is a contribution of this study to the literature. In this way, this study highlights the relationship between different variables taken in the theoretical model of the study to enhance the understanding of the policymakers and the other stakeholders of the healthcare system of diabetes. In addition to it, the significant relationship developed by this study would provide a better understanding for future studies to understand the relationship between different variables and not repeat the same work. This rich contribution to the literature is critical and understandable for future studies to develop the relationship between different variables for working to improve the healthcare system for diabetes patients all over the world.

### 6.2 Practical implications

This study has significant practical implications that are critical to improving the living standard of diabetes patients. To begin with, the study highlights that the responsibility of the doctors and nurses is to be friendly with the patients and provide the appropriate services in the best way to improve their mental ability and eliminate the stigma of mental distress. Similarly, this study highlights that by improving the health care system policies, it would be appropriate for diabetes patients to get treatment from the public and private sector hospitals. Moreover, this study highlights that the emotional distress must be removed from the lives of diabetes patients and they must be motivated for better health services to improve their living standards. In the same way, the responsibility of the hospital administration is to take care of the patients, develop the brand image of the hospital, and provide the best services for the satisfaction of the patients. In this regard, the employees in the service sector must be trained and motivated to perform their responsibilities in the best way and appropriate to the expected standard. Not only, these services would enhance the information of diabetes patients, but these patients would develop their cognitive ability to get the services related to the diseases from the public and private sector hospitals. For improving the access to healthcare, diabetes patients must be motivated by the advanced living standard that provides the best alternative strategies and way for getting the right services.

## 7 Limitations and Future directions

This study aims to investigate the role of diabetic emotional burden and healthcare services as a moderator in the relationship between interpersonal distress, physician & nurse distress, and access to healthcare. The population of this study was the patients with diabetes in different public and private hospitals in Kerala state India. The target population of this study is its limitation. In future studies, the focus should be on a cross-sectional target population, and the subjects from more than one state in India must be considered to validate the results of this study and go with a new dimension to explore future research.

## Abbreviations

T2DM: Type 2 Diabetes mellitus

HCS: Health Care Service

AH: Access to Healthcare

DEB: Diabetes Emotional Burden

ID: Interpersonal Distress

PNRD: Physician and Nurse Distress
